# Satellite tagging of Mediterranean fin whales: working towards the identification of critical habitats and the focussing of mitigation measures

**DOI:** 10.1038/s41598-017-03560-9

**Published:** 2017-06-13

**Authors:** Simone Panigada, Gregory P. Donovan, Jean-Noël Druon, Giancarlo Lauriano, Nino Pierantonio, Enrico Pirotta, Margherita Zanardelli, Alexandre N. Zerbini, Giuseppe Notarbartolo di Sciara

**Affiliations:** 1 Tethys Research Institute, c/o Acquario Civico, Viale G.B. Gadio 2, 20121 Milan, Italy; 2 International Whaling Commission, The Red House, 135 Station Road, Impington, CB24 9NP Cambridge United Kingdom; 3 European Commission, DG Joint Research Centre, Directorate D – Sustainable Resources, Unit D.02 Water and Marine Resources, Via Fermi, TP 051, 21027 Ispra, (VA) Italy; 4 Institute for Environmental Protection and Research - ISPRA, Via V. Brancati 60, 00144 Rome, Italy; 5 School of Mathematics, Washington State University, 14204 NE Salmon Creek Ave, Vancouver, WA 98686 USA; 6 National Marine Mammal Laboratory, Alaska Fisheries Science Center – NOAA, 7600 Sand Point Way N.E., Seattle, WA 98115-6349 USA; 7 Cascadia Research Collective, Olympia, WA USA; 8 Instituto Aqualie, Juiz de Fora, Minas Gerais Brazil

## Abstract

Mediterranean fin whales comprise a genetically distinct population, listed as Vulnerable (VU) in the IUCN Red List. Collisions with vessels are believed to represent the main cause of human-induced mortality. The identification of critical habitats (including migration routes) incorporating satellite telemetry data is therefore crucial to develop focussed conservation efforts. Between 2012 and 2015 thirteen fin whales were equipped with satellite transmitters, 8 in the Pelagos Sanctuary (although two ceased within two days) and 5 in the Strait of Sicily, to evaluate movements and habitat use. A hierarchical switching state-space model was used to identify transiting and area-restricted search (ARS) behaviours, believed to indicate foraging activities. All whales undertook mid- to long-distance migrations, crossing some of the world’s busiest maritime routes. Areas where the animals predominantly engaged in ARS behaviour were identified in both study areas. The telemetry data were compared with results from ecosystem niche modelling, and showed that 80% of tagged whale positions was near (<7 km) the closest suitable habitat. The results contribute to the view that precautionary management should include establishment of a coordinated and dynamic basin-wide management scheme; if appropriate, this may include the establishment of protected areas by specific regional Conventions.

## Introduction

Considerable effort has been devoted to understanding the ecology and conservation status of fin whales (*Balaenoptera physalus*) in the Mediterranean Sea over the past two decades^1–6^. The species is particularly abundant in the Corso-Liguro-Provençal Basin and Gulf of Lions and congregates in the offshore waters of the Pelagos Sanctuary for Mediterranean Marine Mammals^7^, a well-documented summer feeding ground^8^, as well as in the adjacent waters^9^. Winter feeding aggregations have been reported sporadically around the Island of Lampedusa, Strait of Sicily^10^, but no additional information exists on other wintering destinations or on seasonal movements.

Stable isotope and acoustic studies^11–17^ show the presence of two distinct, seasonally overlapping, sub-populations in the Western Mediterranean. ‘True’ Mediterranean fin whales, which are genetically distinct from North Atlantic conspecifics^18, 19^, remain year-round in the central and eastern part of the Basin, while North East North Atlantic (NENA) fin whales, seasonally travel between the North Atlantic Ocean and the south of Spain^20^. This paper focuses on ‘true’ Mediterranean fin whales. Nevertheless, conclusive evidence on the potential input of NENA fin whales to the region - either in the past or at present - remains uncertain.

‘True’ Mediterranean fin whales are listed as Vulnerable (VU) in the IUCN Red List^21^. This population is exposed to several actual and potential threats, with ship strikes being the main cause of human-induced mortality in the Region^22^. Further actual or potential threats include chemical pollution^3, 23, 24^, acoustic disturbance from seismic surveys^25^ and climate change^26^, which may negatively influence the population.

Animal-borne telemetry has been increasingly used during the last decade in a variety of environments and for diverse taxa, contributing important information towards the management of species and their environment, and more in general targeting and informing effective conservation^27^.

ACCOBAMS (the Agreement on the Conservation of Cetaceans in the Black Sea Mediterranean Sea and Contiguous Atlantic Area, under the Convention for Migratory Species) has identified the need to develop a ‘conservation management plan’ for fin whales in the Mediterranean, similar to the approach for such plans developed by the International Whaling Commission (IWC; https://iwc.int/conservation-management-plans). Any such plan requires *inter alia* good knowledge of the distribution, movements and important habitat^28^ of the population in question. Reliable data are essential for the development and success of conservation plans; at the same time it has been recognised that precautionary management, especially for long-lived whales with slow dynamics, may require action before conclusive proof of cause-effect relationships with potential threats^29, 30^. Satellite telemetry can and has provided valuable information on movements and important habitats of cetaceans that can inform management actions^31–34^. This paper provides preliminary information on movements and on the presence of potentially important feeding areas for fin whales in different times of the year, and it also discusses the use of satellite telemetry data to assist in the development of focussed ship strikes mitigation measures, providing indications on the degree of overlap between tagged fin whales and ships (see also supplementary material). While additional data are required to fully address the above mentioned issues, these data contribute to the development and implementation of a proper management and conservation plan for this species in the Mediterranean Sea.

## Methods

### Deployment

**Figure 1:**
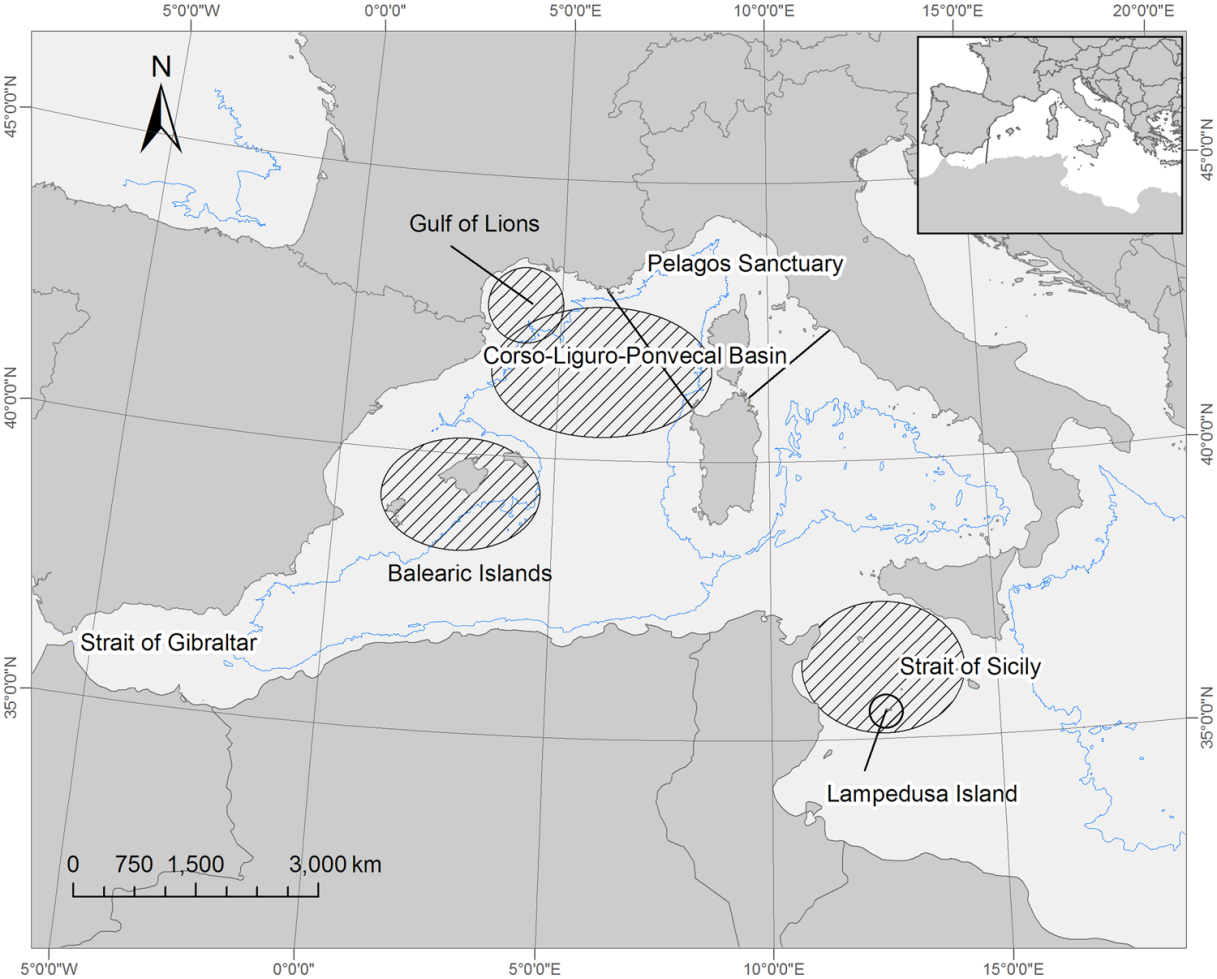
The two study areas selected for the research project: the Pelagos Sanctuary and the Strait of Sicily. The map was produced with ArcGIS Desktop 9.3 (http://www.arcgis.com).

Tag deployment occurred in the Pelagos Sanctuary in September 2012, and around the Island of Lampedusa in March 2013 and 2015 (Fig. 1). Tagging occurred as late in the summer as possible in the Pelagos Sanctuary to gather information outside known summer feeding areas and to observe movements towards winter destinations. In the Strait of Sicily transmitters were deployed in March, when small numbers of whales are known to concentrate for feeding purposes^10^. When possible, individuals equipped with satellite transmitters were visually monitored after tag deployment to qualitatively assess behavioural changes and verify the correct position of the transmitter on the body of the whales. Only adult individuals, visually assessed by the principal investigator, were tagged.

Transdermal (Wildlife Computers molds 177 and 193) and Low Impact Minimally Percutaneous External Electronic (LIMPET, molds 260B and 260C) location-only Argos satellite tags^35–37^ were placed on the dorsum or the dorsal fin of whales. The transdermal tags were deployed with a custom-modified pneumatic line thrower (the Air Rocket Transmitter System – ARTS), while the LIMPET ones with a 150 lb crossbow.

### Analyses

Animals feeding on patchy resources are expected to engage in area-restricted search (ARS) behaviour^38, 39^, characterised by increased turning angles and decreased autocorrelation in direction and speed to maximise searching effort in the most profitable areas. Therefore, Bayesian hierarchical switching state-space models (hSSSM)^40–42^ were fit to Argos location data to differentiate transiting^43^ from ARS behaviours (details in Supplementary Information) and potentially infer preferred habitats. ARS is believed largely to correspond to foraging behaviour, and its occurrence has been used in several studies to identify putative foraging areas^44^.

Although recognising the small sample size, the ARS and tracking data were compared to the results of previous ecological niche modelling (ENM) used to predict the potential feeding habitat of fin whales in the Mediterranean Sea^45^ (see Supplementary Information). This ENM was previously built to integrate knowledge on ecological traits of fin whales (e.g. mobility, feeding strategy) with patterns of selected environmental variables (chlorophyll-a fronts and concentration, water depth) that are thought to be explanatory (or proxy) variables for fin whale distribution. Fin whales were mostly sighted off the continental shelf in the vicinity of chlorophyll-a fronts with low chlorophyll levels (<0.5 mg.m^−3^). Chlorophyll-a fronts are highly dynamic features that have been shown to be hotspots of marine productivity as they remain long enough (weeks to months) to efficiently transfer the energy in marine food webs through zooplankton growth and to attract higher trophic levels (e.g., the Atlantic bluefin tuna^46^ loggerhead sea turtles, albacore tuna^47^, hake recruits^48^).

Using the derived positions of all the whales equipped with satellite transmitter, a Minimum Bounding Geometry (MBG) enclosing each whale location was calculated. The Utilisation Distribution^49, 50^ (UD) was calculated across the MBG to gather information on the spatial extent of animal’s home range, as well as a measure of the spatial intensity of use. UD was calculated through Kernel Density Analysis (KDE) to obtain the likelihood of an animal being present at a given point within the home range^51^. Following the methodology presented by Sprogis *et al*.^52^ KDE accounting for physical barriers to movement was calculated using different toolboxes from the ArcMap 10.1 Software^53^. From KDE values, isopleths (i.e. contours of the UD) were calculated using the Geospatial Modelling Environment^54^ (GME; Version 0.7.2.1) and the statistical software R^55^ to determine Core Home Ranges (50% isopleths; CHR) and Total Home Ranges (90% isopleths; THR)^56, 57^.

As one approach to identifying possible areas of high ship-strike risk, CHRs and THRs were overlaid to density maps of commercial shipping activities^58^ (merchant ships >1000 gross tonnage at sea). Vessel density maps^59–61^ (1 km^2^ raster cells; raster values ranging from 0 to 409; available from https://www.nceas.ucsb.edu/globalmarine/mediterranean) were georeferenced and vectorised in ArcMap 10.1 and high density areas across the MBG were extracted. The percentage of overlap between traffic areas and THR and CHR areas was then calculated.

### Permits

The research was conducted in accordance with the guidelines and authorizations of the competent authorities of France, Italy, and the Principality of Monaco recognising both legal and ethical animal welfare criteria.

Research permits and experimental protocols were issued and approved by:

- Italy: Ministero dell’Ambiente e della Tutela del Territorio e del Mare, Direzione Generale per la Protezione della Natura e del Mare, Divisione II, tutela della biodiversità.
- France: Direction régionale de l’Environnement, de l′Aménagement et du Logement des Provence-Alpes-Côte d’Azur, upon request from the Ministère de l’Écologie, du Développement Durable, des Transports et du Logement.
- Monaco: Département de l′Équipement, de l′Environnement et de l′Urbanisme, Direction des Affaires Maritimes.

## Results

Between 2012 and 2015, thirteen fin whales were tagged with transdermal (average duration = 45.6 days, SD = 51.0) or LIMPET tags (mean = 29.6 days, SD = 10.3). Eight transmitters (5 transdermal and three LIMPET) were deployed in 2012 in the Pelagos Sanctuary, while 3 transdermal and 2 LIMPET tags were deployed in the Strait of Sicily, in 2013 and 2015, respectively (Table 1).

**Table 1:** Summary of tag performances.

Tag type	Deployment date	Tag performances			hSSSM results		
		Tag PTT number	Deployment area	Days of transmission	Transit (%)	ARS (%)	Uncertain (%)
Transdermal	07/09/2012	112707	Pelagos Sanctuary	32*	—	—	—
Transdermal	08/09/2012	112697*a*	Pelagos Sanctuary	142	10	75	15
		112697*b*			15	58	27
Transdermal	09/09/2012	112716*a*	Pelagos Sanctuary	83	10	10	80
		112716*b*			4	75	21
Transdermal	11/09/2012	112709	Pelagos Sanctuary	2^†^	—	—	—
Transdermal	17/09/2012	112708*a*	Pelagos Sanctuary	81	0	78	22
		112708*b*			9	70	21
LIMPET	22/09/2012	102223	Pelagos Sanctuary	18	2	70	28
LIMPET	22/09/2012	102221	Pelagos Sanctuary	35	9	55	36
LIMPET	22/09/2012	102224	Pelagos Sanctuary	22	0	68	32
Transdermal	03/03/2013	111868	Strait of Sicily	8	0	70	30
Transdermal	04/03/2013	120938	Strait of Sicily	4	12	63	25
Transdermal	11/03/2013	120942	Strait of Sicily	13	11	70	19
LIMPET	14/03/2015	87776	Strait of Sicily	29	15	63	22
LIMPET	14/03/2015	87780	Strait of Sicily	44	20	64	16

For each individual fin whale, the percentage of locations where the behavioural state was classified as transiting, ARS or uncertain (based on Jonsen *et al*.’s^62^ conservative cut-off values) is reported. The uncertain category indicates locations where the model could not distinguish conclusively between the two behavioural modes, either because of intermediate characteristics of the movement process or of large Argos error (see Supplementary Information for additional details). When the temporal gap between consecutive locations was longer than four days, tracks were split into separate segments for the analysis. These are indicated here with the letter *a* and *b*, respectively. *No location, only transmissions were received from this tag thus it was not included in the analysis; ^†^The hSSSM analysis was not performed for this tag given its relatively short duration.

### Corso-Liguro-Provençal Basin

**Figure 2:**
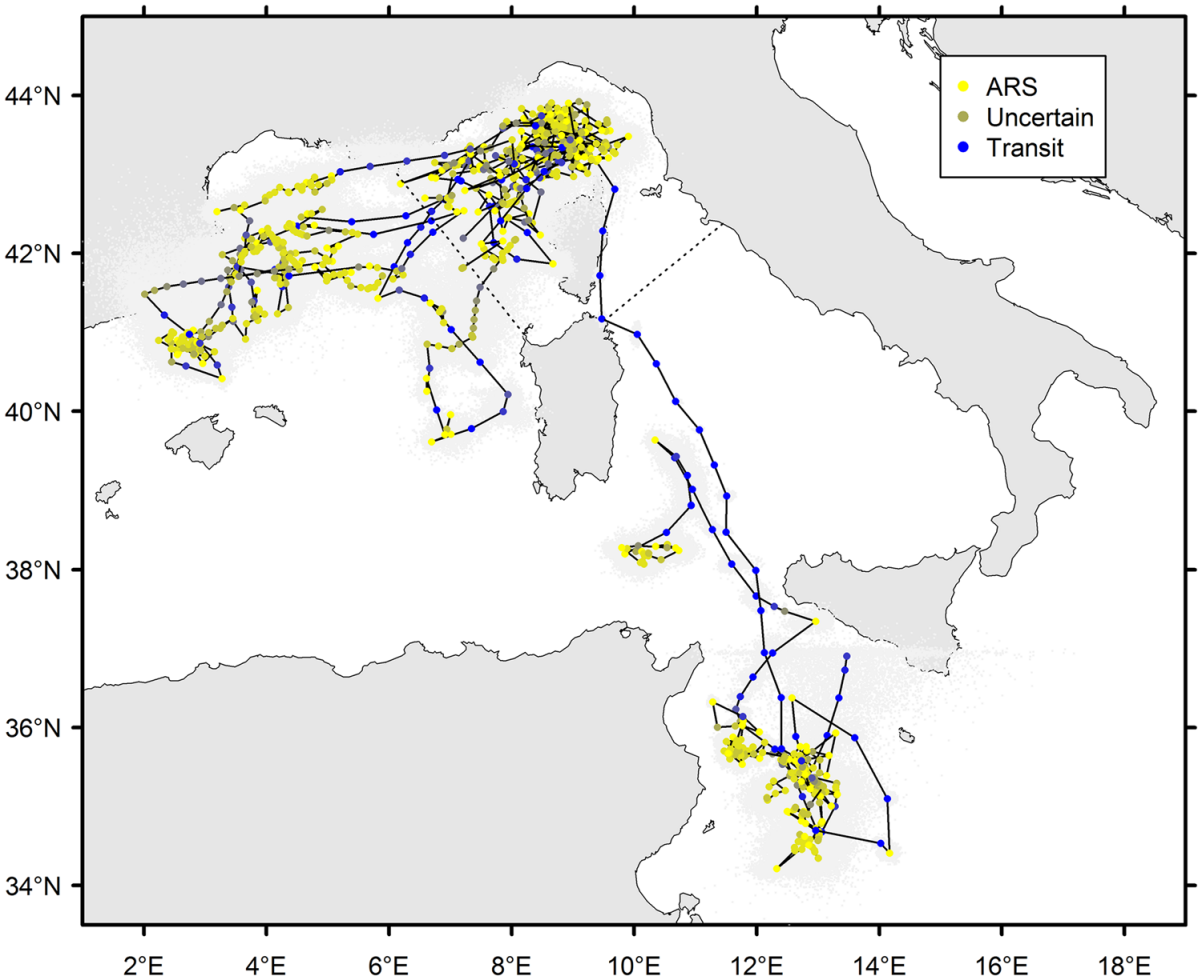
Telemetry tracks of fin whales tagged in the Ligurian Sea in 2012 in the Strait of Sicily in 2013 and 2015, reconstructed from the posterior estimates of the parameters of the hSSSM, and inferred transiting, ARS and uncertain behaviours (based on Jonsen *et al*.’s^62^ conservative cut-off values). The light grey points represent model uncertainty around each location, as indicated by the latitude and longitude values at each MCMC iteration. The map was generated using R software, version 3.2.3 (R Core Team, 2015) with packages PBSmapping (Schnute *et al*. 2015) and rgdal (Bivand *et al*. 2016). R Foundation for Statistical Computing, Vienna, Austria (www.R-project.org).

Two out of eight tags deployed in 2012 transmitted for less than 2 days and are not further considered here. The remaining tags revealed consistent movements within the Corso-Liguro-Provençal Basin (Fig. 2), with whales mainly remaining in the waters of the Pelagos Sanctuary and adjacent waters until transmissions stopped. Two individuals (PTT: 112716, 112708, Fig. 2) moved towards the Gulf of Lions and the Balearic Islands at the end of October 2012, remaining in this area for approximately one month before transmissions stopped.

The hSSSM showed fin whales predominantly engaged in ARS behaviour (66%), with potential feeding areas being revealed within the Corso-Liguro-Provençal Basin and towards the Balearic Islands (Fig. 2). Behavioural state estimates indicated a 27% of locations classified as uncertain under the conservative cut-off values proposed by Jonsen *et al*.^62^ (see Supplementary material). Inspection of the diagnostic plots and posterior distributions suggested this may reflect the small spatial scale of the animals’ displacement, compared to the accuracy and frequency of the Argos satellite fixed locations^63^. These locations could also correspond to transition periods between the two behaviours or to short periods of directed movement within longer ARS intervals^62^, but these sources of uncertainty are confounded^63^.

**Figure 3:**
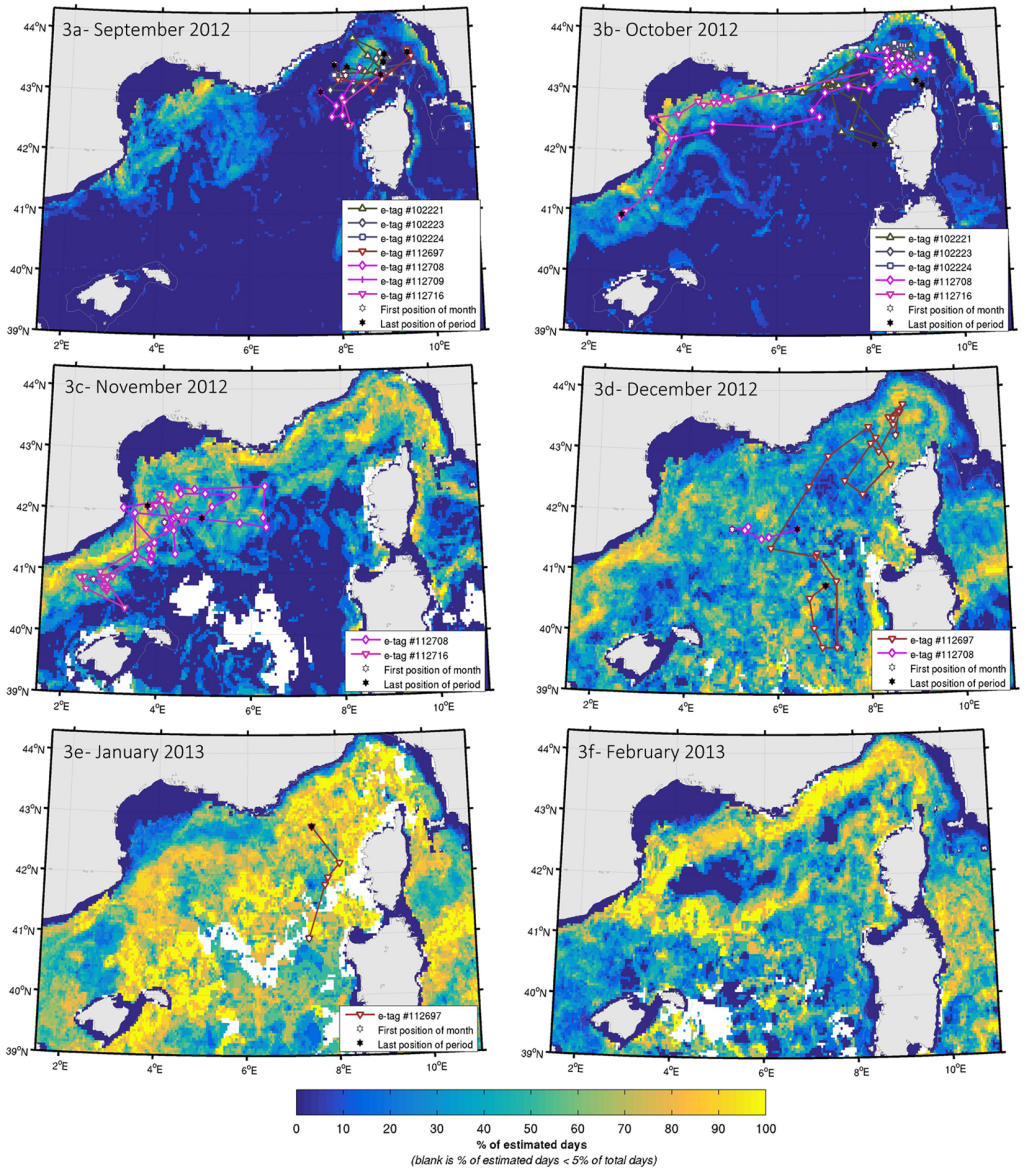
Seven Argos satellite derived positions overlaid on monthly mean potential feeding habitat (frequency of occurrence from September 2012 to February 2013). Note that only one position per day is shown. The potential habitat is derived from the daily detection of chlorophyll-a front, a range of surface chlorophyll-a content and a minimum water depth (see Supplementary materials for details). The 200 m depth contour is shown. The maps were prepared off-line using a commercial software package: MATLAB and Statistics Toolbox Release 2015b, The MathWorks, Inc., Natick, Massachusetts, United States (www.mathworks.com).

The overlap between the whales’ satellite-derived positions and potential foraging habitats obtained from the ENM in the Corso-Liguro-Provençal Basin is shown in Fig. 3 (see discussion).

### Strait of Sicily

Animals tagged in the Strait of Sicily remained around the Island of Lampedusa for a significant portion of the time they were tracked (March) (Fig. 2). While tags deployed in 2013 did not last long enough for tracking mid- or long-range movements, the longevity of tags improved in 2015 and movements towards the southern coast of Sicily and northern Tunisia were observed. Shorter tracks could be included in the analysis because of the hierarchical nature of the state-space model, which integrated information from longer tracks to classify behavioural states. Most of the whales sighted off Lampedusa in 2013–2015 were observed actively feeding at the surface on large swarms of krill, most likely of the species *Nyctiphanes couchii* ^10^ (personal observations).

The hSSSM analysis revealed a high rate of ARS behaviour (65%), suggesting several potential feeding areas in the Strait of Sicily (Fig. 2), including the one previously reported around the Island of Lampedusa^10^. Two other areas, one south-east of Lampedusa and another closer to the coast of Tunisia, may also represent important habitat for fin whales. In fact, after spending 19 days in the Strait of Sicily, one fin whale (PTT 87776) moved north towards the Southern Tyrrhenian Sea and the east coast of Sardinia Island. Then it headed back towards the south of Sardinia, between Tunisia and the Island of Lampedusa where it remained for a few days engaging in intense feeding before transmissions were interrupted.

After remaining in the Sicily Strait area for a month, the second whale (PTT 87780) moved northwards to the area of the Pelagos Sanctuary, remaining for eight days north of Corsica Island before moving south-westward. The hSSSM analysis highlighted the presence of potential feeding areas in both the Strait of Sicily and the Ligurian Sea, corroborating previous knowledge and further supporting the importance of these areas for the ecology of the species in the Region (Fig. 3).

**Figure 4:**
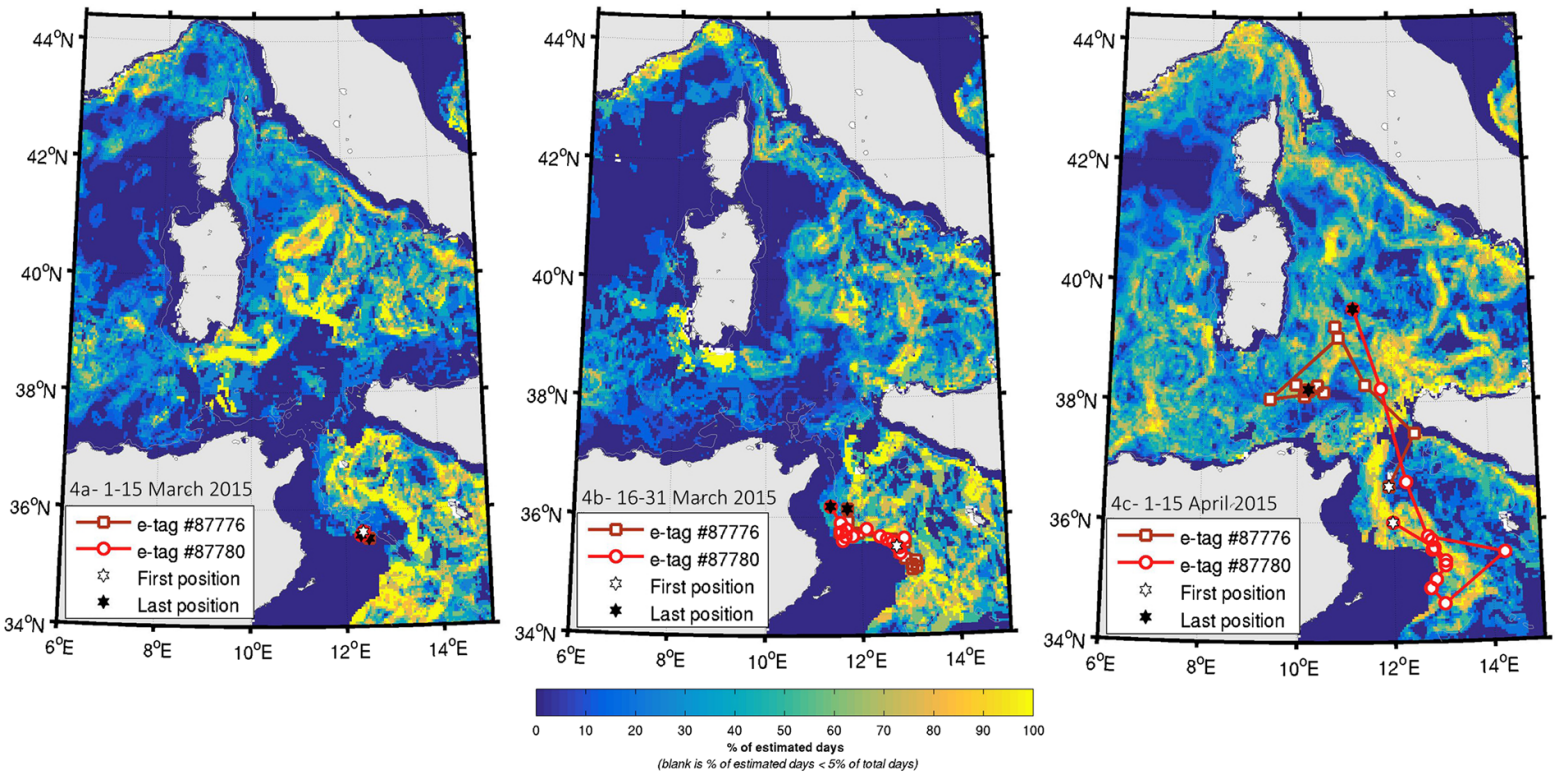
Two Argos satellite derived positions overlaid on fortnights mean potential feeding habitat (March-April 2015). Note that only one position per day is shown. The potential habitat of fin whale is derived from the daily detection of chlorophyll-a fronts, a range of surface chlorophyll-a content and a minimum water depth (see Supplementary materials for details). The 200 m depth contour is shown. The maps were prepared off-line using a commercial software package: MATLAB and Statistics Toolbox Release 2015b, The MathWorks, Inc., Natick, Massachusetts, United States (www.mathworks.com).

The overlap between the whales’ satellite-derived positions and potential foraging habitats obtained from the ENM in the Strait of Sicily and in the South-western Tyrrhenian Basin, between Sicily and Sardinia, is shown in Fig. 4 (see discussion).

### Traffic and whale movements

**Figure 5:**
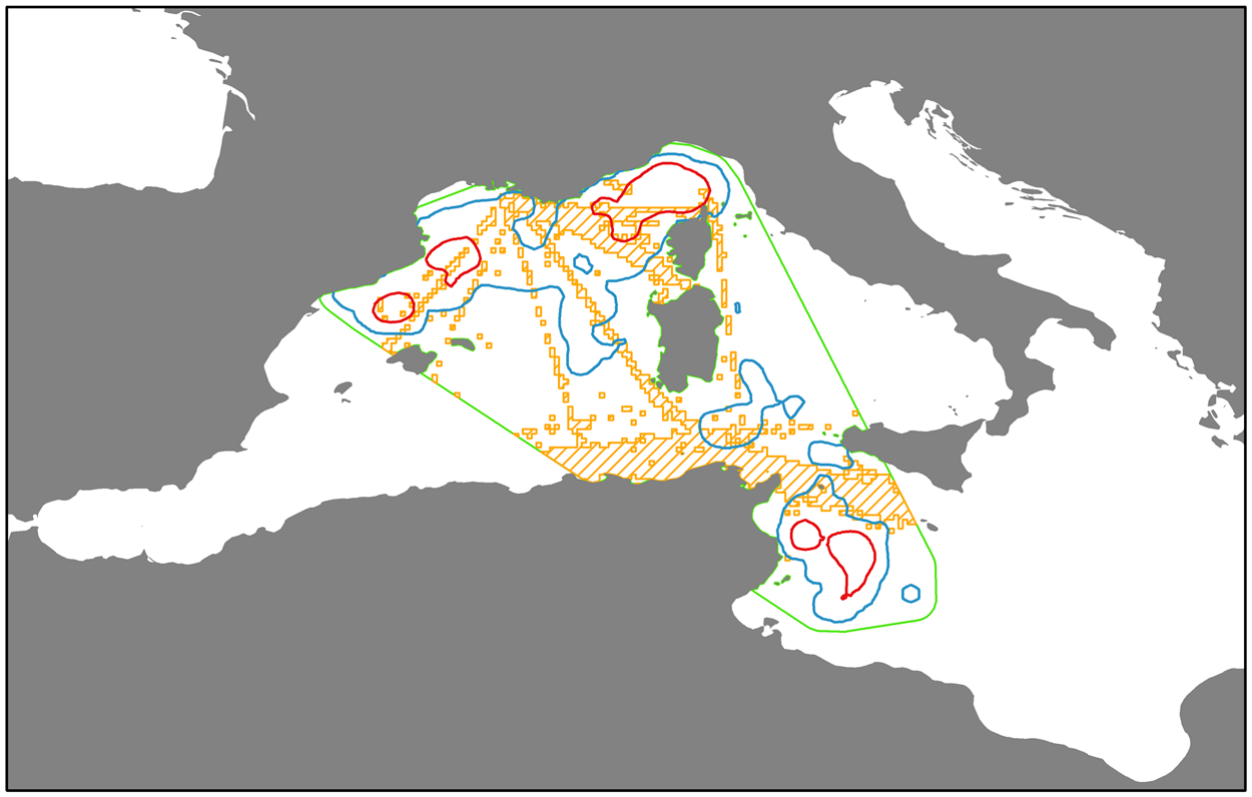
Overlapping of the 90% total home range areas and 50% core home range with regions of high traffic usage. Green dashed line represents the minimum bounding geometry enclosing all whale locations. Orange dashed area corresponds to areas of high traffic densities. Kernel derived total home ranges (90% isopleths) in blue and core home ranges (50% isopleths) in red. The map was produced with ArcGIS Desktop 9.3 (http://www.arcgis.com).

When considering both the 90% total home range and 50% core home range areas, the 17.4% of the former and 14.0% of the latter overlap with areas of high traffic volumes within the minimum bounding geometry (MBG) (Fig. 5).

## Discussion

Satellite telemetry data such as that from Mediterranean fin whales in the Pelagos Sanctuary and in the Strait of Sicily between 2012 and 2015 – further complemented with spatial modelling, for example with results from ecological niche modelling and from aerial surveys^5^ – can provide an important contribution with other data (e.g. from cetacean abundance surveys and information on human activities) to begin to prioritise and help develop conservation actions^27, 64–68^ (e.g. suggesting areas where conservation actions such as reduced ship speed^58, 69, 70^ or regulation of whale watching activities) within the species’ range, both in summer and winter seasons. Understanding why whales are where they are when they are is a vital component of developing effective mitigation measures and focussing where they are needed. This is an important task but it must be recognised that for long-lived, wide-ranging and difficult-to-study species such as whales, this will be inevitably a cumulative process over a number of years. We fully recognise that this will require the collection of more satellite derived data from fin whales, as well as building up of additional data on explanatory variables and improved modelling (e.g. ref. 71) from a number of sources, but for illustrative purposes of this paper, comparison of the tracks of the whales with the results of predictive feeding areas from one modelling approach^40^ for the same time periods has been presented. This does not imply that that this particular ENM approach and the use of chlorophyll-a data is the best proxy for fin whale feeding habitat, but it rather provides a useful start to an ongoing process, as does work on spatial modelling from ship and aerial surveys (e.g. refs 5, 72 and 73).

### Corso-Liguro-Provençal Basin

The overlap between the whales’ satellite-derived positions and potential foraging habitats obtained from the ENM in the Corso-Liguro-Provençal Basin, is shown in Fig. 3.

The ENM used chlorophyll-a fronts as a proxy for food availability^45^. Based upon this, the potential feeding habitat for fin whales in the north-western Mediterranean Sea during the 2012 study period expanded from a limited extent during late summer in the Corso-Liguro-Provençal Basin (Fig. 3a,b) to most of that area from November 2012 (Fig. 3c) to the entire western Mediterranean by January 2013 (Fig. 3d,e), prior to tracing the general circulation of the Western Mediterranean in February 2013 in the northern part of the basin (Fig. 3f). The surface area of suitable habitat for the period 2003–2014 ranged from 7 to 8% of the western Mediterranean Sea in July-September (4–6% for 2012) to 27% in January 2003–2014 (37% for 2013). The analysis showed that 80% of tagged whale positions (n = 415) were closer than 7 km to the closest suitable habitat, which is consistent with the overall habitat model performance of 8 km (n = 1287)^45^. The spatial dispersion of the six whales from the Ligurian Sea in September 2012 to most of the northern basin in the following months matches the habitat expansion, with clear phases of transit when successive positions are distant, and ARS behaviour otherwise (Fig. 2). Feeding habitat contraction within Pelagos during summer, and successive expansion during winter, could provide insights into the mechanisms driving fin whales’ concentration there in summer and dispersion later^20^.

Krill in the Ligurian Sea has been reported in spring, summer and autumn with strandings of *M. norvegica* swarms along the coast of western Liguria, southern France and Monaco during the winter months^74, 75^. Nonetheless, fin whales’ presence in the area drastically drops during winter^5^, with animals primarily occurring during the early summer and engaging in feeding^8^. This seasonal occurrence of fin whales in the Ligurian Sea has been related to the fact that, despite the overall year-round presence of krill as possible prey, potential feeding habitats require high density swarms and not simply high average concentrations; these are substantially more spread over the basin in winter^45^, leading whales to migrate outside the well-known summer feeding grounds to take advantage of higher concentration of food in other areas^76^. The tagged whales remained in the Pelagos Sanctuary and adjacent waters longer in the season than described in the available literature, engaging in ARS behaviour for a significant portion of time (66%). Due to the errors associated with ARGOS fixes and the small scale of the displacement in this region, behaviour was classified as uncertain (under the conservative criteria proposed by Jonsen *et al*.^62^) in almost a third of the locations^63^. The poor quality of ARGOS locations contributed to these moderate levels of uncertainty in the behavioural classification by the state-space model. While disentangling ARGOS error (intrinsic in our data) from the uncertainty associated with the behavioural process would only be possible with an independent index of behavioural state (which is not currently available), our conclusions regarding the whales’ ecology are not affected by the locations classified as uncertain.

Notwithstanding these limitations, the whales clearly showed a complex movement pattern in the area. It is interesting to note that the longer transiting movements corresponded with areas that were not identified as potential feeding habitat by the ENM (Fig. 3). This also supports the view that whales persisted in using the Pelagos Sanctuary at the end of summer due to favourable conditions allowing for prolonged feeding.

The results presented here, even if based on a small sample size, emphasize that potentially important fin whale habitat extends westward of the Pelagos Sanctuary^9, 77^, well within the north-western Mediterranean Pelagic Ecosystems Ecologically or Biologically Significant Marine Area (EBSA, https://chm.cbd.int/database/record?documentID=204125), identified by the Convention on Biological Diversity (CBD). Moreover,  previous research along the eastern borders of the Sanctuary^78^, as well as spatial modelling of aerial survey data, showed a longitudinal gradient in the occurrence of fin whales within the northern Pelagos Sanctuary, predicting higher densities west of Sardinia, primarily in deep offshore waters^79^.

### Strait of Sicily

The overlap between the whales’ satellite-derived positions and potential foraging habitats obtained from the ENM in the Strait of Sicily and in the South-western Tyrrhenian Basin, between Sicily and Sardinia, is shown in Fig. 4.

The ENM and tracking results from the Strait of Sicily in 2015 (Fig. 4) showed that potential feeding habitat extended well beyond the near-shore waters of the Island of Lampedusa^10^. Whales remained in this area for almost a month after tagging, engaging in ARS behaviour 65% of the time, prior to moving northwards. In addition to being a hotspot for Mediterranean biodiversity (https://chm.cbd.int/database/record?documentID=204108), the Strait of Sicily also represents the main deep-water vessel traffic channel connecting the Eastern and Western basins of the Mediterranean, with severe traffic volumes between the Suez Canal and the Strait of Gibraltar^80^ (see Fig. S1 in the Supplementary Information). This may have potentially serious impacts to fin whales, both with respect to generated underwater noise^81–83^ and ship strike risk^22^.

### General observations on movements/migration

The observed longitudinal movements of fin whales tagged in the Ligurian Sea in the late summer and the latitudinal migration recorded in early spring, support the hypothesis that the whales summering in the north-western Mediterranean Sea travel southward towards the winter feeding grounds in the Strait of Sicily, and possibly towards non identified breeding areas in the Southern Mediterranean Sea^8, 12^. One additional hypothesis arising out of our limited information that requires additional research effort is that whales would later move northbound towards the Pelagos Sanctuary and adjacent waters during the early- mid-spring, following the marked feeding habitat concentration in the area described by Notarbartolo di Sciara *et al*.^20^.

The results of the hSSSM analysis for one whale tagged in the Strait of Sicily (PPT 87780), and, to a lesser extent, of the whales tracked in the Ligurian Sea, suggest that, despite the presence of potential feeding areas along their tracks, whales may travel consistently between different locations without suspending their movements to engage in ARS behaviour. Although clearly a sample size of one is insufficient, this pattern has been observed for fin whales in other areas^44^ and is consistent with the idea that for energetic reasons, whales typically exploit areas of high concentration of food resources, without capitalising on small patches of prey^38^. The ENM takes into account the size of chlorophyll-a fronts - the hypothesis is that the larger the productivity of the frontal feature and the longer its stability over time, the higher the probability that dense aggregations of prey will form, attracting feeding fin whales.

The movements between the waters of the Strait of Sicily and the Ligurian Sea indicate that at least some of the whales that visit the Strait of Sicily in winter will congregate later in spring and summer in the North-Western Mediterranean. This supports the hypothesis of seasonal movements of Mediterranean fin whales between the two areas, probably related at least in part to feeding^8, 12^. The seasonal presence of fin whales further to the east, including in the Ionian^84^ and Southern Adriatic Seas^85^, can also be explained by more complex searching for prey concentrations across the central Mediterranean.

The connection between whales seen off Lampedusa and those that spend the summer in Pelagos Sanctuary – also witnessed by photo-identification data and suggested by aerial survey data^5, 86^ - illustrates large and defined seasonal movements of fin whales through the Mediterranean. It is therefore evident that a substantial portion of the Central and North-Western Mediterranean plays an important role in the ecology of the species. By extension, especially in light of the high level of anthropogenic stressors affecting the area, it is also an important area for conservation efforts. The northbound migration routes shown here reveal that the animals move through areas with high human activity. Overlap of whale presence with such activity gives rise to increased exposure to threats, such as ship strikes, where at present no protection or mitigation schemes are in place. This new information, whilst preliminary, highlights the need for early consideration of a comprehensive basin-wide mitigation scheme to complement any national measures, in conjunction with additional research to provide a fuller understanding of whale distribution and behaviour at appropriate geographical and temporal scales.

The movements described here, assuming that chlorophyll-a is a reasonable proxy for potential feeding areas, appears to match the dynamics of the habitat. It suggests that whales have the ability to track food resources over time in order to exploit seasonally and spatially restricted habitats during the peaks of prey abundance and perhaps ensure prolonged access to higher-quality foraging opportunities. Thus, the movements observed within the north-Western Mediterranean Sea, between the Gulf of Genoa and the Balearic Islands, as well as between the Strait of Sicily and the Pelagos Sanctuary, is an effective response to resource fluctuations, both within and across seasons and areas.

Although of limited sample size, the data show a synchrony between the start of the northbound migration and the shifting in the occurrence of potential feeding habitats. For example, two whales remained in the area off Lampedusa in March, when the probability of feeding habitat is high in the Strait of Sicily and very low in the Tyrrhenian and Ligurian Seas. As feeding habitat decreased in the Strait of Sicily and increased in the South Tyrrhenian Sea during the first half of April, both whales started to move northbound towards richer areas. The whale with the remaining transmitter in the second half of April pursued its northward migration to the area of the Pelagos Sanctuary as the habitat became more productive, before transmissions stopped. Movements of both whales demonstrate the switch of favourable habitat occurrence in the first half of April from the area south of Sicily to the western Mediterranean Sea. In particular, the second whale went directly to the summer feeding grounds (900 km apart in a straight line distance) in about five days.

### Conservation implications

Ship strikes represent the main cause of concern for the conservation of fin whales across the Mediterranean basin^87^. This issue is being addressed at the regional and international levels through a joint effort by ACCOBAMS, the Pelagos Sanctuary Agreement and the International Whaling Commission (IWC). These organizations work towards the identification of high-risk areas where to apply and test mitigation actions. Among the measures currently in place in other areas of the world, mainly implemented through the International Maritime Organization (IMO), there are Areas To Be Avoided (ATBA), Particularly Sensitive Sea Areas (PSSA) and the establishment or the shifting of Traffic Separation Schemes (TSS). A proposal by France is currently envisaging the establishment of a PSSA within the Pelagos Sanctuary borders, while the presented results would argue in favour of a wider area, possibly encompassing more critical habitats for fin whales, such as CBD’s ‘north-western Mediterranean Pelagic Ecosystems’ EBSA. The data collected here, once supplemented by additional deployments and integrated with data from other whale studies and information on human activities such as vessel traffic, are informative for the implementation and assessment of these initiatives. The approach illustrated here (Fig. 5), identifying areas used by fin whales and areas of high traffic volumes, is one that can be improved and developed with additional data.

We recognise that the present findings alone are insufficient to identify robustly the most important areas for fin whales. However, they do provide an important start to this task and support the importance of beginning political efforts to develop an effective multi-national conservation management plan for this species with a designated action plan to address actual and potential threats. Approaches to consider include the establishment of a seasonal/dynamic focussed protection regime (e.g. a Marine Protected Area or a Specially Protected Area of Mediterranean Importance (SPAMI) under the auspices of the Barcelona Convention). Static MPAs often fail to encompass the dynamic features that many pelagic and mobile species exploit^88^. Hence, despite substantial practical and legal challenges, spatially and temporally dynamic protection schemes, intended to change their location and size based on habitat characteristics, species occurrence and movements, as well as water column features, could be effective in protecting highly mobile species, provided that they are accompanied by the necessary research to develop robust models of whale occurrence at the appropriate spatial and temporal scales^89–91^.

## Conclusions

The Pelagos Sanctuary and its surrounding bodies of water represent the major summer feeding grounds for the species^8^ and host critical feeding habitats also for a large portion of the autumn and early winter months. It is therefore crucial that mitigation of detrimental activities, such as maritime traffic (see Fig. S1 in the Supplementary Information), and overall actions to warrant the protection and conservation of fin whales and their critical habitats extend beyond the present boundaries of the Sanctuary.

Satellite telemetry combined with habitat modelling is one important tool to assess critical and highly used habitats of fin whales in the Mediterranean Sea and important migration routes. Such information is essential to prioritise mitigation of human-induced threats, including, for example, ship strikes.

As models become more robust - and more data are collected - the results can feed into coordinated and dynamic management schemes^92^ – as foreseen by the Marine Spatial Planning (MSP) - to try to effectively protect fin whales in the Mediterranean. Information on the temporal and geographical distribution and abundance of fin whales (and thus fin whales’ critical habitats) are also required for identification of Important Marine Mammal Areas (IMMAs), which could lead to the proposal for the establishment of Specially Protected Areas of Mediterranean Importance (SPAMIs) under the framework of the Barcelona Convention. It is therefore recommended that further studies, such as the one proposed and described here, be conducted also in other targeted areas of the Mediterranean Basin such as the Ionian Sea and the North African coasts, to provide a more complete picture of the whales’ distribution and movements within the Mediterranean and the presence of their critical habitats.

## Electronic supplementary material

Supplementary information
