# Supplementary material for: Satellite tagging of Mediterranean fin whales: working towards the identification of critical habitats and the focussing of mitigation measures
Source: Sci Rep. 2017 Jun 13;7:3365. doi: 10.1038/s41598-017-03560-9 (PMC5469747; doi:10.1038/s41598-017-03560-9)
Supplement: Supplementary file 1 — Supplementary information [file 41598_2017_3560_MOESM1_ESM.pdf]

Supplementary Information

Satellite tagging of Mediterranean fin whales: working towards the identification of critical habitats and the focussing of mitigation measures

SIMONE PANIGADA[1*], GREGORY P. DONOVAN[2], JEAN-NOËL DRUON[3], GIANCARLO LAURIANO[4], NINO PIERANTONIO[1], ENRICO PIROTTA[5], MARGHERITA ZANARDELLI[1], ALEXANDRE N. ZERBINI[6,7,8], GIUSEPPE NOTARBARTOLO DI SCIARA[1]

[1] Tethys Research Institute, c/o Acquario Civico, Viale G.B. Gadio 2, 20121 Milan, Italy

[2] International Whaling Commission, The Red House, 135 Station Road, Impington, CB24 9NP, Cambridge, United Kingdom

[3] European Commission, DG Joint Research Centre, Directorate D – Sustainable Resources, Unit D.02 Water and Marine Resources, Via Fermi, TP 051, 21027 Ispra (VA), Italy

[4] Institute for Environmental Protection and Research - ISPRA, Via V. Brancati 60, 00144 Rome, Italy

[5] School of Mathematics, Washington State University, 14204 NE Salmon Creek Ave, Vancouver, WA 98686, United States

[6] National Marine Mammal Laboratory, Alaska Fisheries Science Center – NOAA, 7600 Sand Point Way N.E., Seattle, WA 98115-6349 USA

[7] Cascadia Research Collective, Olympia, WA, USA

[8] Instituto Aqualie, Juiz de Fora, Minas Gerais, Brazil

[*] Correspondence to panigada69@gmail.com

Habitat modelling

Compared to the habitat modelling described in Druon et al. 2012[1], where frontal features were shown to play a major aggregation role for that species, the favourable feeding habitat was presently traced by the identification of sea surface chlorophyll-a fronts only and not in combination with the satellite-derived SST fronts. Indeed, infrared data (to derive SST) were found to be more unstable from day to day than the optical data (to derive surface chlorophyll-a content). Overall, the potential feeding habitat derived on a daily basis uses the productive frontal features of chlorophyll-a, a preferred range of surface chlorophyll-a concentration and a minimum water depth. The used optimized set of parameters using chlorophyll-a (CHL) from MODIS-Aqua sensor has the following values:

- linear increase of daily habitat from 0.3 to 1 in the range of CHL gradient from 0.00086 to 0.0052 $mgCHL.m^{-3}.km^{-1}$, and 1 above the latter value,

- $0.11 < \text{CHL concentration} < 0.50\ mg.m^{-3}$

- Water depth > 90 m

Bayesian hierarchical switching state-space models

The package *bsam* for R was used for the analysis[2,3]; *bsam* fits hierarchical switching state-space models (hSSSM) using Markov Chain Monte Carlo (MCMC) simulation methods implemented via JAGS[4]. Using a hierarchical approach allowed us to include short tracks, since information for behavioural classification is obtained by analysing these together with longer deployments[5]. We chose an 8 h time step, which was larger than 80% of the observed time steps. This avoided excessive extrapolation over unobserved portions of a whale's track, while offering a sufficient temporal resolution to characterise switches in behaviour. Poor quality locations of class "Z" were removed prior to modelling to facilitate convergence. Tracks with gaps longer than four days

between consecutive locations were split into separate segments. Two MCMC chains were run in parallel for 120,000 iterations. 100,000 iterations were discarded as burn-in, and 1 every 20 observations was retained for the remaining 20,000 samples, to reduce autocorrelation. Convergence was assessed by inspecting trace, autocorrelation and posterior density plots. Point estimates and uncertainty for model parameters were derived from 2,000 samples from the joint posterior distribution (1,000 samples per chain). The behavioural state of whale k in each time step t (b_{kt}) is a binary variable that can take value of 1 (transiting) or 2 (ARS). Following Jonsen et al.[6], the behavioural state at each location was classified as ARS if the posterior mean of b_{kt} was greater than 1.75, as transiting if the mean was smaller than 1.25, and as uncertain otherwise. We followed this conservative approach as a way to openly acknowledge the uncertainty associated with the behavioural classification[6]. Uncertain classification indicates instances where the model could not distinguish conclusively between the two behaviours due to intermediate characteristics of the animal's movement process or to large Argos location error relative to the scale of the movements[6,7]. The two sources of uncertainty are confounded[7] and could only be conclusively disentangled using an independent measure of behavioural state, which is not available for our study animals.

We inspected the plots of the posterior tracks, which suggested that uncertain locations corresponded to short periods of directed movement within longer ARS phases, often in association with infrequent or unprecise positioning by Argos. The posterior distributions of model parameters also showed that there was no issue with convergence or mixing for the transition probabilities between states, and that the persistence parameters were well separated for the two states. These results suggest that the model could identify the two states correctly and that these did not show overlapping characteristics, indicating that the observation error associated with Argos locations was the most probable cause of the moderate levels of uncertain behavioural classification. In addition, we considered two tracks (87776 and 87780) for which the scale of transit movements was larger than Argos error. As expected, these two tracks were characterized by a lower proportion of uncertain locations. We ran the model separately for the two tracks and found that the

proportion of uncertain locations remained low. This provides additional support to the conclusion that the uncertainty in behavioural classification in other tracks mainly arose from Argos observational error relative to the scale of transit.

Table S1 – Summary of the hSSSM analysis by tagging area and overall.

**Tagging area**	**hSSSM results**		
	**Transit (%)**	**ARS (%)**	**Uncertain (%)**
Pelagos Sanctuary	7	66	27
Strait of Sicily	16	65	19
overall	10	66	24

AIS and Shipping data

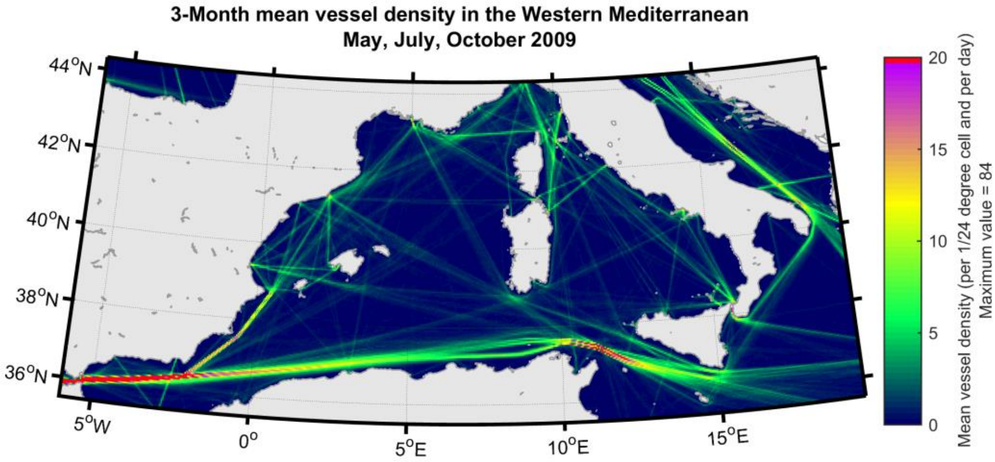

Fig. S1 - Mean traffic density for May, July and October 2009 in the Western Mediterranean Sea (in number of vessels per day and cells of 1/24 degree) from AIS data[8]. The JRC has an open access policy and document: https://ec.europa.eu/jrc/en/publication/eur-scientific-and-technical-research-reports/jrc-data-policy

References

1. Druon, J. *et al.* Potential feeding habitat of fin whales in the western Mediterranean Sea: an environmental niche model. *Mar. Ecol. Prog. Ser.* **464,** 289–306 (2012).
2. Bestley, S., Jonsen, I. D., Hindell, M. A., Guinet, C. & Charrassin, J.-B. Integrative modelling of animal movement: incorporating in situ habitat and behavioural information for a migratory marine predator. *Proc. R. Soc. Lond. B Biol. Sci.* **280,** 20122262 (2013).
3. R Development Core Team. *R: A language and environment for statistical computing. R Foundation for Statistical Computing, Vienna, Austria. ISBN 3-900051-07-0, URL http://www.R-project.org/.* (2013).
4. Plummer, M. JAGS: A program for analysis of Bayesian graphical models using Gibbs sampling. in *Proceedings of the 3rd International Workshop on Distributed Statistical Computing* (2003).

5. Jonsen, I. Joint estimation over multiple individuals improves behavioural state inference from animal movement data. *Sci. Rep.* **6,** (2016).
6. Jonsen, I. D., Myers, R. A. & James, M. C. Identifying leatherback turtle foraging behaviour from satellite telemetry using a switching state-space model. *Mar. Ecol. Prog. Ser.* **337,** 255–264 (2007).
7. Breed, G. A., Costa, D. P., Goebel, M. E. & Robinson, P. W. Electronic tracking tag programming is critical to data collection for behavioral time-series analysis. *Ecosphere* **2,** art10 (2011).
8. Vaes, T. & Druon, J.-N. *Mapping of potential risk of ship strike with fin whales in the Western Mediterranean Sea. A scientific and technical review using the potential habitat of fin whales and the effective vessel density*. (European Commission Joint Research Centre Institute for the Protection and Security of the Citizen).
